# Effects of *Lactobacillus plantarum*-Fermented Feed on Growth and Intestinal Health in *Haliotis discus hannai*

**DOI:** 10.3390/microorganisms13071603

**Published:** 2025-07-08

**Authors:** Ling Ke, Chenyu Huang, Song Peng, Mengshi Zhao, Fengqiang Lin, Zhaolong Li

**Affiliations:** 1 The Research Institute of Biotechnology, Fujian Academy of Agricultural Sciences, Fuzhou 350013, China; keling@faas.cn; 2 Institute of Animal Husbandry and Veterinary Medicine, Fujian Academy of Agricultural Sciences, Fuzhou 350013, China; nimoy88@163.com (C.H.); pengsong@faas.cn (S.P.); 13375001253@163.com (M.Z.); linfengqiang@faas.cn (F.L.)

**Keywords:** *Haliotis discus hannai*, *Lactobacillus plantarum*, fermented feed, growth, intestinal health

## Abstract

This study multidimensionally investigates the comprehensive effects of *Lactobacillus plantarum* (LP)-fermented feed on growth performance, intestinal health, and metabolic regulation in Pacific abalone (*Haliotis discus hannai*). The results demonstrate that LP fermentation significantly alters feed’s physical properties and nutritional profile, softening texture, increasing viscosity, and emitting an acidic aroma. Notably, it enhanced contents of cis-9-palmitoleic acid, α-linolenic acid (ALA), and functional amino acids (GABA, L-histidine, and L-asparagine), indicating that fermentation optimized ω-3 fatty acid accumulation and amino acid profiles through the modulation of fatty acid metabolic pathways, thereby improving feed biofunctionality and stress-resistant potential. Further analyses revealed that fermented feed markedly improved intestinal morphology in abalone, promoting villus integrity and upregulating tight junction proteins (ZO-1, Claudin) to reinforce intestinal barrier function. Concurrently, it downregulated inflammatory cytokines (TNF-α, NF-κB, IL-16) while upregulating anti-inflammatory factors (TLR4) and antioxidant-related genes (NRF2/KEAP1 pathway), synergistically mitigating intestinal inflammation and enhancing antioxidant capacity. Sequencing and untargeted metabolomics unveiled that fermented feed substantially remodeled gut microbiota structure, increasing Firmicutes abundance while reducing Bacteroidetes, with the notable enrichment of beneficial genera such as *Mycoplasma*. Metabolite profiling highlighted the significant activation of lipid metabolism, tryptophan pathway, and coenzyme A biosynthesis. A Spearman correlation analysis identified microbiota–metabolite interactions (such as Halomonas’ association with isethionic acid) potentially driving growth performance via metabolic microenvironment regulation. In conclusion, LP-fermented feed enhances abalone growth, immune response, and aquaculture efficiency through multi-dimensional synergistic mechanisms (nutritional optimization, intestinal homeostasis regulation, microbiota–metabolome crosstalk), providing critical theoretical foundations for aquafeed development and probiotic applications in aquaculture.

## 1. Introduction

Haliotis discus hannai, a pivotal subspecies within the genus Haliotis (family Haliotidae) [1], predominantly inhabits rocky substrates across tropical to temperate marine zones [2]. Within these habitats, specific water temperature, transparency, and light intensity constitute critical ecological determinants, and wild populations establish stable ecological niches in algae- and kelp-rich environments [3], exhibiting trophic specialization through macroalgal consumption. Despite these specific habitat requirements in the wild, H. discus hannai is recognized as the cornerstone species in China’s abalone aquaculture industry. H. discus hannai is the most extensively farmed abalone species in northern China, especially in the Shandong, Liaoning, and Fujian provinces. It constitutes > 70% of China’s farmed abalone output due to its rapid growth, superior flesh quality, and adaptability to controlled environments [4]. However, industrial-scale farming faces dual challenges: escalating biosecurity risks from pathogenic infections, and systemic nutritional deficits.

Vibriosis and pseudomonad-induced pathologies (such as pustule disease and abdominal rupture) have emerged as primary biosecurity threats [5,6,7,8], with elevated water temperatures during summer exacerbating pathogen proliferation, immunosuppression, and mass mortality events. Concurrently, high-density farming amplifies pathogen transmission via hydrodynamic vectors. Traditional algal-based feeds fail to meet protein–lipid requirements, while artificial formulations, despite storage stability advantages, suffer from suboptimal cellulose digestibility and imbalanced amino acid profiles, collectively impairing growth performance and elevating production costs [9]. These intertwined constraints necessitate innovative technological interventions.

Probiotics, as bioactive microbial preparations, demonstrate multifaceted therapeutic potential through host–microbiome crosstalk modulation. Their tripartite mechanisms encompass the following: gut microbiota regulation via the competitive exclusion of pathogens (Vibrio harveyi) and short-chain fatty acid (SCFA)-mediated enteric homeostasis [10]; nutrient metabolism enhancement through the enzymatic hydrolysis of recalcitrant feed components (cereal proteins/polysaccharides), achieving 8–34% weight gain improvement [11,12,13]; and immunomodulation via microbial-associated molecular patterns (MAMPs) activating pattern recognition receptors (PRRs), thereby triggering TLR4/NRF2/KEAP1 signaling cascades to suppress pro-inflammatory cytokines (TNF-α, IL-16) [14]. Notably, Shewanella-supplemented diets reduce *V. harveyi* mortality from 77% to 27% [15], while *Bacillus* strains enhance hemocyte vitality and apoptosis resistance [16]. Furthermore, probiotics exhibit an environmental bioremediation capacity, degrading 99% of nitrites and ammonia nitrogen in aquaculture systems [17]. *Lactobacillus plantarum* (LP) was selected as the focal probiotic agent in this study due to its (1) well-documented safety (GRAS/QPS status) and stability in feed matrices, (2) robust enzymatic repertoire (proteases, carbohydrases) that enables the efficient degradation of complex plant-derived antinutrients, (3) potent SCFA (particularly lactate) production capacity, which acidifies the gut lumen to inhibit Vibrio spp. colonization, and (4) proven immunostimulatory effects in aquatic species via the upregulation of antioxidant pathways and antimicrobial peptide expression [17]. Its resilience to industrial processing (lyophilization, pelleting) further enhances its practical applicability in aquaculture feed production.

The synergistic integration of probiotic engineering with feed biotechnology presents transformative potential. LP-fermented feeds degrade antinutritional factors, enrich γ-aminobutyric acid (GABA) and α-linolenic acid (ALA) profiles [18], and activate the tryptophan metabolism. Probiotic formulations achieve mortality reduction [19] and reshape gut microbiomes by elevating Firmicutes abundance, fostering nutrient assimilation and immune priming [20]. The primary goal of this investigation is to rigorously evaluate the efficacy of a novel LP-fermented feed in (1) augmenting growth performance and feed utilization efficiency, (2) enhancing resistance against Vibrio harveyi challenges, and (3) modulating key immune-physiological and metabolic responses (e.g., microbiome structure, antioxidant status). This paradigm not only truncates traditional 3–4 year cultivation cycles, but enables precision health management through microbiome–metabolome axis elucidation (Halomonas–hydroxyethyl sulfonate metabolic coupling).

## 2. Materials and Methods

### 2.1. Preparation and Quality Analysis of Fermented Feed

#### 2.1.1. Bacterial Culture Preparation and Fermentation Process

The LP-NDMJ-4 bacterial suspension (viable count: 7 × 10^8^ CFU/mL, isolated and preserved by our research center, Fuzhou, China) was mixed with soybean meal substrate (crude protein ≥ 45%, Shandong Sanwei Soybean Protein Co., Ltd., Linyi, China) at a 5% (*v*/*v*) inoculum ratio, and the moisture content was adjusted to 15%. The mixture was loaded into breathable membrane fermentation feed bags (specifications: 30 cm × 40 cm, air permeability: 5000 mL/(m^2^·h); Qingdao Haida Biotechnology Group Co., Ltd., Qingdao, China) and fermented at 37 °C for 3 days in a constant temperature incubator (SPX-250B, Shanghai Yiheng Scientific Instruments Co., Ltd., Shanghai, China). Post-fermentation, samples were collected for quality assessment.

#### 2.1.2. Feed Quality Assessment

##### Sensory Evaluation and pH Measurement

The sensory attributes (aroma, color, and appearance) of fermented and unfermented feeds were comparatively scored. For pH measurement, 3 g of sample was mixed with 30 mL of ultrapure water (Milli-Q system, Merck Millipore, Darmstadt, Germany), incubated at 37 °C with shaking (THZ-98A thermostatic shaker, Shanghai Zhicheng Analytical Instruments Co., Ltd., Shanghai, China) for 30 min, and analyzed using a pH meter (FE28, Mettler Toledo, Greifensee, Switzerland).

##### Nutritional Profiling (Gas Chromatography–Mass Spectrometry, GC-MS Analysis)

Samples were extracted with isopropanol (High Performance Liquid Chromatography HPLC grade, Sigma-Aldrich, St. Louis, MO, USA)-n-hexane (HPLC grade, Sinopharm Chemical Reagent Co., Ltd., Shanghai, China) (2:3 *v*/*v*, containing 0.2 mg/L internal standard), followed by liquid nitrogen grinding (JXFSTPRP-CL grinding instrument, Shanghai Jingxin Technology, Shanghai, China), ice-water bath ultrasonication (KQ-500DE, Kunshan Shumei Ultrasonic Instrument Co., Ltd., Kunshan, China) for 5 min, and centrifugation at 12,000× *g* for 15 min at 4 °C (Centrifuge 5424R, Eppendorf, Hamburg, Germany). The supernatant was dried under nitrogen gas (TurboVap LV, Biotage, Uppsala, Sweden), derivatized with methanol:trimethylsilyl diazomethane (1:2 *v*/*v*, Sigma-Aldrich), redissolved in n-hexane, and analyzed via GC-MS (7890B-5977B, Agilent Technologies, Santa Clara, CA, USA).

##### Amino Acid Analysis (UHPLC-MS/MS)

Samples were extracted with acetonitrile (HPLC grade, Merck, Darmstadt, Germany)–methanol (HPLC grade, Sigma-Aldrich)–water (Milli-Q) (2:2:1 *v*/*v*, containing isotope-labeled internal standards), subjected to liquid nitrogen grinding, ultrasonication, and centrifugation. The supernatant was analyzed using UltraHigh Performance Liquid Chromatography tandem mass spectrometry (UHPLC-MS/MS) (Vanquish Horizon-Q Exactive HF-X, Thermo Fisher Scientific, Waltham, MA, USA) for amino acid composition.

### 2.2. Experimental Animals and Husbandry

#### 2.2.1. Experimental Design

A total of 300 juvenile abalone (H. discus hannai) with similar body weights (19 ± 1.5 g) were obtained from Fujian Xiapu Rixing Aquaculture Cooperative (Ningde, China) and divided into three groups: the CK group, fed an unfermented basal diet (formulation: 15% fish meal, 40% kelp powder, 40% soybean meal, 5% spirulina; all ingredients purchased from Qingdao Haida Biotechnology Group); the HD group, fed on natural kelp feed (Fujian Xiapu Kelp Aquaculture Base, Ningde, China); and the LP group, fed L. plantarum NDMJ-4 fermented feed. Each group consisted of 100 individual abalone, with a 90-day rearing period. All animal experiments were conducted in accordance with the requirements of the Animal Ethics Committee of the Fujian Academy of Agricultural Sciences Institute of Animal Husbandry and Veterinary Medicine (MTLLSC 2024-001-2).

#### 2.2.2. Rearing Conditions

Water temperature (22–28 °C) was monitored using a temperature logger (HOBO MX2201, Onset Computer, Bourne, MA, USA), pH (7.0–8.1) measured using a pH meter (FE28, Mettler Toledo), and dissolved oxygen (6.0–8.5 mg/L) was calibrated using a portable dissolved oxygen meter (Pro20, YSI, Yellow Springs, OH, USA). Feed was administered every 5 days (500 g per cage) using aquaculture cages (40 cm × 30 cm × 12 cm, Fujian Xiapu Fishing Gear Factory, Ningde, China).

#### 2.2.3. Sample Collection

At the end of the trial, 20 abalones per group were randomly selected. Body weight was measured using an electronic balance (ME104E, Mettler Toledo), and shell length/width were determined using a vernier caliper (500-196-30, Mitutoyo, Kawasaki, Japan). Intestinal tissues were divided into two portions: one portion from 10 abalones per group stored at −80 °C (DW-HL668 ultra-low temperature freezer, Zhongke Meiling, Hefei, China) for DNA extraction and subsequent microbiota analysis, and the other fixed in 4% paraformaldehyde (Servicebio, Wuhan, China) for HE staining.

### 2.3. Growth Performance Evaluation

The following indices were calculated:

Weight Gain Rate (WGR):

$$\frac{Final\;weight-Initial\;weight}{Initial\;weight}\times 100\%$$

Survival Rate (SR):

$$=\frac{Number\;of\;survivors}{Initial\;number}\times 100\%$$

Specific Growth Rate (SGR)

$$=\frac{Final\;weight-Initial\;weight}{Experimental\;days}\times 100\%$$

Feed Conversion Ratio (FCR):

$$=\frac{Total\;feed\;intake}{Total\;weight\;gain}\times 100\%$$

### 2.4. Intestinal Histomorphology and Barrier Function Analysis

#### 2.4.1. Intestinal Tissue Fixation and HE Staining

Abalone intestinal tissues were rinsed with physiological saline (Sinopharm Chemical Reagent Co., Ltd., Shanghai, China), and 1 cm segments were fixed in 4% paraformaldehyde (pH 7.4, Servicebio, Wuhan, China) at 4 °C for 24 h. Tissues were dehydrated in graded ethanol (70%, 80%, 90%, 100%; analytical grade, Sinopharm), cleared in xylene (analytical grade, Sinopharm), embedded in paraffin wax (Leica Biosystems, Wetzlar, Germany), and sectioned at 5 μm thickness. Sections were stained with hematoxylin (Harris method, Servicebio) for 5 min, differentiated in 1% acid ethanol, blued in running water, and counterstained with eosin (Servicebio) for 1 min. Neutral resin (Servicebio) was used for mounting. Intestinal villus structure, goblet cell distribution, and inflammatory infiltration were observed under a Nikon ECLIPSE Ci microscope (Nikon, Tokyo, Japan), with images captured using the NIS-Elements D 5.11 system (Nikon).

#### 2.4.2. Intestinal Barrier-Related Gene Expression Analysis

Intestinal tissues (20 mg) were homogenized in liquid nitrogen, lysed with 1 mL NucleoZOL (Macherey-Nagel, Düren, Germany), and incubated for 5 min. After adding 0.2 mL chloroform (HPLC grade, Sigma-Aldrich), samples were vortexed for 15 s and centrifuged at 12,000× *g* for 15 min at 4 °C (Eppendorf Centrifuge 5424R). The aqueous phase was mixed with isopropanol (Sigma-Aldrich) to precipitate RNA. RNA pellets were washed twice with 75% ethanol, dissolved in DEPC-treated water (Sigma-Aldrich), and quantified for purity (A260/A280 > 1.8, NanoDrop 2000, Thermo Fisher Scientific) and concentration (Qubit 4.0 Fluorometer, Thermo Fisher). Reverse transcription was performed using the Yeasen Hifair^®^ III 1st Strand cDNA Synthesis Kit (Yeasen, Shanghai, China) in a 20 μL reaction: 4 μL 5× gDNA Digester Mix, 4 μL 5× Hifair^®^ Buffer, 1 μL Oligo(dT) primer, 1 μL Random Hexamers, and 1 μL Enzyme Mix. The reaction conditions were 25 °C for 5 min, 55 °C for 15 min, and 85 °C for 5 min. qRT-PCR was conducted in a 10 μL system containing 0.8 μL cDNA, 0.5 μM primers (Table 1), and 5 μL 2× SYBR Green Master Mix (Yeasen) on a Roche LightCycler 96 qPCR system (Roche, Basel, Switzerland) with the following program: 94 °C for 30 s (pre-denaturation), 40 cycles of 94 °C for 5 s, 60 °C for 15 s, and 72 °C for 10 s, followed by melt curve analysis (65–95 °C, 0.5 °C/s). Relative gene expression was calculated using the 2−ΔΔCt method with GAPDH as the reference, and three technical replicates were performed per group.

### 2.5. Gut Microbiota Diversity and Composition Analysis

#### 2.5.1. Microbial Genomic DNA Extraction and Sequencing

Total DNA was extracted from intestinal contents (50 mg) using the CTAB/SDS method. Samples were homogenized with 500 μL lysis buffer (100 mM Tris-HCl, 40 mM EDTA, pH 8.0, Sigma-Aldrich) and 0.5 g glass beads (0.1 mm diameter, BioSpec Products, Bartlesville, USA), followed by incubation with 20 μL proteinase K (20 mg/mL, Qiagen, Hilden, Germany) at 65 °C for 1 h. After chloroform:isoamyl alcohol (24:1, Sigma-Aldrich) extraction and isopropanol precipitation, DNA pellets were washed with 75% ethanol and dissolved in TE buffer (10 mM Tris-HCl, 1 mM EDTA, pH 8.0, Sigma-Aldrich). DNA integrity was assessed via 1% agarose gel electrophoresis (Biowest, Barcelona, Spain), quantified using Qubit, and the V3-V4 region of 16S rDNA was amplified with primers 338F (ACTCCTACGGGAGGCAGCAG) and 806R (GGACTACHVGGGTWTCTAAT) (Sangon Biotech, Shanghai, China). PCR products were purified with AMPure XP beads (Beckman Coulter, Brea, CA, USA) and sequenced on the Illumina NovaSeq 6000 platform (Illumina, San Diego, CA, USA) in PE250 mode (≥50,000 reads per sample).

#### 2.5.2. Bioinformatics Analysis

Raw sequencing data were quality-controlled using Fastp v0.23.2 (BGI, Shenzhen, China) (Q20 ≥ 90%, removal of non-specific sequences). OTUs were clustered at 97% similarity using UPARSE v11 (Robert C. Edgar, Berkeley, CA, USA), chimeras were removed with UCHIME, and taxonomic annotation was performed using the SILVA v138 database (https://www.arb-silva.de/). Alpha diversity indices (Shannon, Chao1, Simpson) were calculated via QIIME2 (Greg Caporaso, Flagstaff, AZ, USA). Beta diversity was analyzed using PCoA based on Unweighted Unifrac distances (phyloseq package in R, R Foundation R.4.3.8, Vienna, Austria). LefSe analysis (linear discriminant analysis (LDA) > 4, Kruskal–Wallis test *p* < 0.05) identified differential taxa, visualized using the R package ggplot2 (3.5.0).

### 2.6. Non-Targeted Metabolomic Analysis on Gastric and Intestinal Content Samples

#### 2.6.1. Metabolite Extraction and LC-MS Detection

Liquid nitrogen-ground intestinal tissues (100 mg) were extracted with 500 μL pre-cooled 80% methanol (containing 0.1% formic acid, HPLC grade, Sigma-Aldrich), vortexed for 1 min (Vortex-Genie 2, Scientific Industries, Bohemia, NY, USA), ultrasonicated in an ice bath (40 kHz, KQ-500DE, Kunshan Shumei, Kunshan, China) for 10 min, and centrifuged at 15,000× *g* for 20 min at 4 °C (Eppendorf). The supernatant (300 μL) was diluted with 120 μL ultrapure water (Milli-Q system, Merck Millipore) to 53% methanol, centrifuged again, and 200 μL was injected for analysis. Chromatographic separation was performed on a Thermo Scientific Hypersil Gold C18 column (2.1 mm × 100 mm, 1.9 μm, Thermo Fisher) with mobile phase A (0.1% formic acid in water) and B (0.1% formic acid in methanol). The gradient program was 0–1.5 min (2% B), 3 min (85% B), and 10 min (100% B), followed by equilibration at 2% B. Metabolites were detected using a Thermo Q Exactive HF-X mass spectrometer (Thermo Fisher) with a HESI ion source (positive/negative modes), spray voltage 3.5 kV (+)/2.8 kV (−), full scan range *m*/*z* 70–1050 (resolution: 120,000), and MS/MS in Top 10 mode.

#### 2.6.2. Metabolite Identification and Pathway Analysis

Raw data were converted to mzXML format using ProteoWizard (ProteoWizard Foundation, Cambridge, MA, USA). Peak extraction, alignment, and normalization were performed using XCMS (Scripps Research, San Diego, CA, USA; parameters: bw = 5, ppm = 10, minfrac = 0.5). Metabolites were annotated via HMDB (http://www.hmdb.ca/, accessed on 26 June 2025), KEGG (https://www.kegg.jp//, accessed on 26 June 2025), and METLIN (Scripps Research) databases (mass error < 5 ppm, retention time deviation < 0.2 min, MS/MS similarity > 80%). Differential metabolites were subjected to KEGG pathway enrichment analysis (MetaboAnalyst 5.0, https://www.metaboanalyst.ca//, accessed on 26 June 2025; *p* < 0.05, FDR-corrected), visualized as volcano plots, heatmaps, and pathway bubble charts using the R package ggplot2(3.5.0).

### 2.7. Correlation Analysis

Correlation analysis among 19 gut microbial taxa, 19 differential metabolites, and key seawater indicators was performed as follows: Microbial taxa were identified via 16S rRNA sequencing of abalone gastrointestinal contents based on abundance variations. Metabolites were quantified via metabolomics, while seawater indicators were assessed using environmental monitoring tools (such as Lianchuan Cloud platform). Spearman’s rank correlation analysis evaluated monotonic relationships between variables, visualized via heatmaps to highlight interaction patterns.

### 2.8. Bioinformatics and Statistical Analysis

Data were analyzed using GraphPad Prism 9.0 (GraphPad Software, San Diego, CA, USA). One-way ANOVA was applied, and the results are expressed as mean ± standard deviation. A *p*-value < 0.05 was considered statistically significant.

## 3. Results

### 3.1. Analysis of Feed Quality Before and After Fermentation

#### 3.1.1. Sensory Evaluation of Fermented Feed

**Figure 1:**
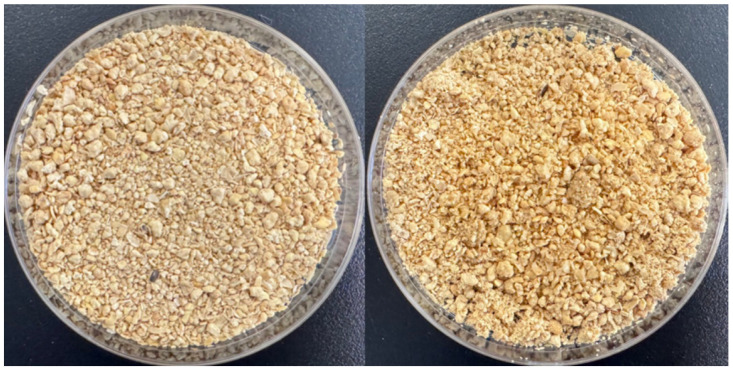
Granular state of soybean meal before and after fermentation. (**A**) Pre-fermented soybean meal. (**B**) Fermented soybean meal.

As shown in Table 1 and Figure 1, the color of LP-fermented feed exhibited minimal variation compared to the control group, both presenting a brownish-yellow hue. Post-fermentation, the aroma of soybean meal transitioned from its original bean cake scent to a distinctive sour and alcoholic fragrance. The addition of fermentation broth increased feed moisture content, resulting in a softer texture with minor agglomeration, although no significant pH alteration was observed (pH 6.5 vs. 6.7).

#### 3.1.2. Impact of Probiotic Fermentation on the Nutritional Profile of Soybean Meal

As indicated in Table 2, LP fermentation notably enhanced specific fatty acid contents. The LP group demonstrated a two-fold increase in cis-9-palmitoleic acid (106.85 ± 21.37 mg/kg vs. 48.32 ± 9.18 mg/kg in CK) and a 14% elevation in α-linolenic acid (ALA) content (292.6 ± 55.59 mg/kg vs. 256.6 ± 33.36 mg/kg). However, docosahexaenoic acid (DHA) levels decreased by 11.8% (66.47 ± 13.29 mg/kg vs. 75.33 ± 10.55 mg/kg), warranting further investigation into fermentation-induced metabolic pathway alterations. A marginal increase was observed in cis-11,14,17-eicosatrienoic acid (2.47 ± 0.3 mg/kg vs. 1.97 ± 0.28 mg/kg).

Concerning saturated fatty acids (Table 3), caprylic acid (C8:0) content decreased by 20.53% in the LP group, while lauric acid (C12:0), tridecanoic acid (C13:0), and myristic acid (C14:0) exhibited dramatic increases of 357.14%, 163.64%, and 51.42%, respectively.

#### 3.1.3. Amino Acid Profile Modifications Induced by Probiotic Fermentation

Fermentation significantly elevated amino acid levels in the LP group compared to CK(Table 4). The most pronounced increases were observed in L-histidine (+38.69%), L-asparagine (+36.10%), γ-aminobutyric acid (+32.88%), and L-Arginine (+29.51%).

### 3.2. Production Performance

Production performance refers to the comprehensive evaluation of abalone growth, survival rate, and feed utilization efficiency during aquaculture, serving as a critical indicator for assessing rearing conditions, feed quality, and management efficacy. As detailed in Table 5, the growth performance of abalone varied significantly across feed treatment groups. The weight gain rate in the LP group (421%) was significantly higher than that in the CK (201%) and HD (263%) groups.

The feed conversion ratio (FCR), a key metric for evaluating feed utilization efficiency, was calculated as 2633.12 for the LP group, lower than both the CK (1586.37) and HD (1205.36) groups. In terms of survival rate, the LP group achieved 61.66%, outperforming the CK group (45.33%), but remaining lower than the HD group (69%). Notably, while the total weight gain rate of the LP group (263%) substantially exceeded that of the CK group (201%), it was markedly lower than the HD group’s 421%.

### 3.3. Determination of Abalone Quality

The experiment compared changes in amino acid content in abalone muscle among the HD group (kelp-based feed), LP group (LP-fermented feed), and CK group (basal feed) (Table 6). The results reveal that the LP group exhibited higher concentrations of most amino acids in abalone muscle compared to both the HD and CK groups. Specifically, histidine, phenylalanine, and cystine levels in the LP group were 0.21 g/kg, 0.41 g/kg, and 0.11 g/kg, respectively. These values represented increases of 0.10 g/kg, 0.09 g/kg, and 0.10 g/kg compared to the CK group (0.11 g/kg, 0.32 g/kg, and 0.09 g/kg), and increments of 0.03 g/kg, 0.04 g/kg, and 0.10 g/kg relative to the HD group (0.18 g/kg, 0.37 g/kg, and 0.01 g/kg). These findings indicate that fermented feed enhances the umami-related components in abalone muscle.

### 3.4. Effects of LP-Fermented Feed on Intestinal Morphology of Abalone

**Figure 2:**
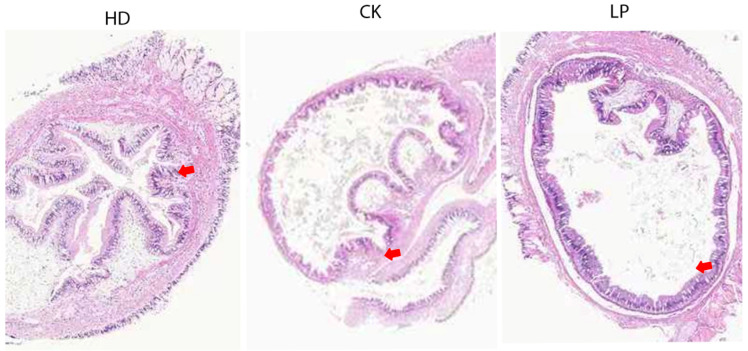
Section of the intestinal tissue of Abalone discus humphead (HE staining, 40×). HD: natural feed group; CK: control group; LP: LP-fermented group.

A comparative analysis of intestinal tissue structure (Figure 2) revealed significant morphological differences in abalone intestines across feed treatment groups. In the HD group, the intestinal architecture remained relatively intact, characterized by well-developed villi and a continuous mucosal layer. The abundant villous structure effectively expanded the nutrient absorption surface area, thereby enhancing dietary nutrient utilization and promoting growth. These findings suggest that kelp-based feed may exert protective effects on intestinal integrity, supporting barrier function and nutrient assimilation. In contrast, the CK group exhibited disorganized intestinal folds with shortened, sparse villi and localized structural damage, indicating the limited benefits of basal feed on intestinal health. Such compromised morphology likely reduced nutrient absorption efficiency, ultimately impairing growth performance.

The LP group demonstrated markedly improved intestinal integrity compared to CK, with significantly elongated and denser villi and no observable structural defects. Although slightly inferior to the HD group, the LP group’s intestinal morphology surpassed that of CK, highlighting that LP NDHJ-4 fermentation enhanced digestive capacity and intestinal health in *H. discus hannai* through structural optimization.

### 3.5. Effects of LP-Fermented Feed on Intestinal Barrier Function, Cytokines, and Antioxidant Factors in Abalone

**Figure 3:**
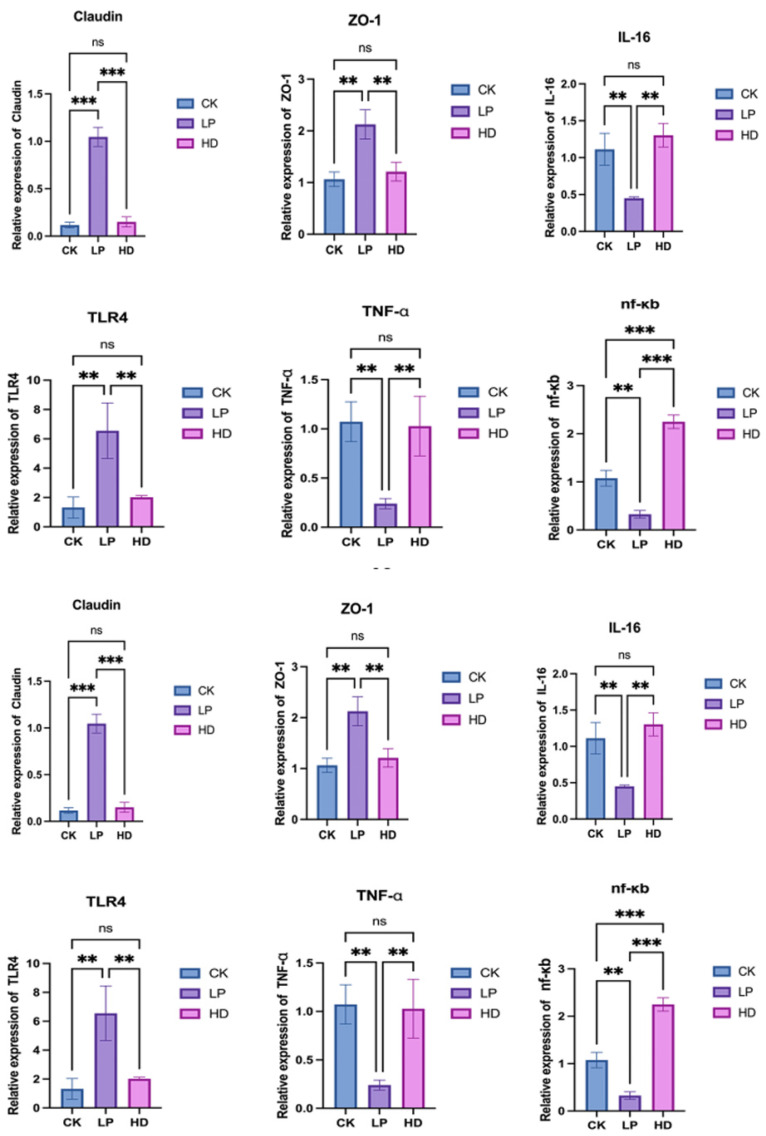
Effects of feeding different diets on the intestinal tissue and cytokine mRNA expression of H. discus hannai. **: *p* < 0.01; ***: *p* < 0.001; ns = not significant (*p* ≥ 0.05).

A comparative analysis of intestinal tight junction protein mRNA expression across feed treatment groups (Figure 3) revealed distinct molecular responses. The LP group exhibited significantly elevated Claudin mRNA expression compared to both CK and HD groups (*p* < 0.001), with no significant difference observed between CK and HD (ns). Similarly, ZO-1 mRNA expression was markedly higher in the LP group than in CK and HD (*p* < 0.01), indicating enhanced tight junction assembly and intestinal barrier integrity in the fermented feed group.

Inflammatory and immune-related cytokine profiling demonstrated the regulatory effects of LP treatment. While TLR4 mRNA expression showed no difference between CK and HD, it was significantly upregulated in LP (*p* < 0.01). Conversely, TNF-α mRNA expression in LP decreased sharply compared to CK and HD (*p* < 0.01), with no intergroup difference between CK and HD. Notably, NF-κB mRNA levels were lowest in LP, while HD displayed the highest expression (*p* < 0.001 vs. both groups), and CK levels exceeded LP (*p* < 0.01). Additionally, IL-16 mRNA expression in LP was reduced by 42% compared to CK and HD (*p* < 0.01), further suggesting that fermented feed mitigates inflammatory signaling.

An antioxidant pathway analysis revealed that Keap1 mRNA expression peaked in CK, but was suppressed in LP and HD (*p* < 0.001). Although Nrf-2 mRNA levels showed no difference between CK and LP, HD exhibited significantly higher Nrf-2 expression than CK (*p* < 0.05), highlighting divergent antioxidant activation mechanisms.

### 3.6. Effects of Fermented Feed on the Intestinal Microbiota of Abalone

#### 3.6.1. OTU Distribution and Diversity Analysis

**Figure 4:**
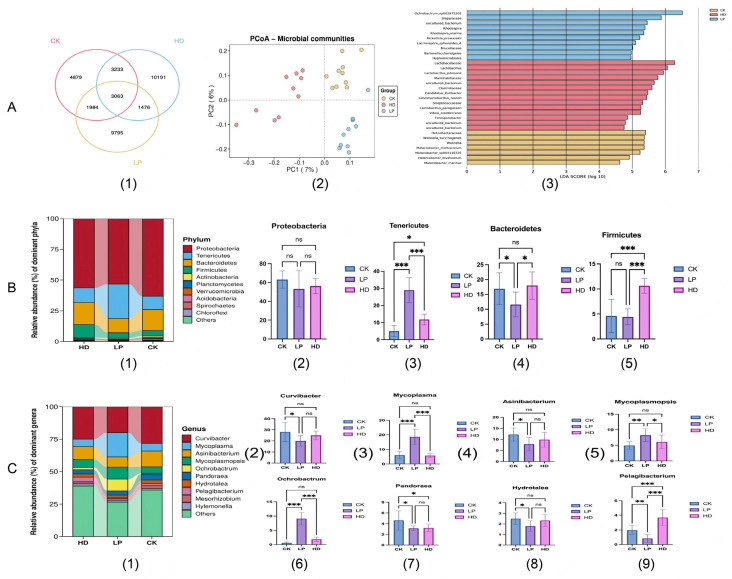
Effects of fermented feed on gut microbiota composition in abalone. (**A1**) Venn diagram of OTU distribution. (**A2**) Analysis of Beta diversity. (**A3**) Analysis of LDA effect size of the different groups. (**B1**–**B5**) Changes in microbial flora at the portal level. (**C1**–**C9**) Changes in microbial flora at the genus level. * *p* < 0.05; **: *p* < 0.01; ***: *p* < 0.001; ns = not significant (*p* ≥ 0.05).

The Venn diagram (Figure 4) illustrates the similarity of operational taxonomic unit (OTU) composition among three abalone intestinal microbiota groups. A total of 46,574 OTUs were identified across all groups, with 3063 OTUs shared among all three groups. Specifically, the CK group contained 13,159 OTUs (4879 unique), the HD group exhibited the highest richness with 17,963 OTUs (10,191 unique), and the LP group comprised 16,318 OTUs (9795 unique). This hierarchical distribution indicates that the HD group demonstrated the highest OTU richness, followed by the LP and CK groups.

Alpha diversity indices were analyzed to evaluate microbial community characteristics (Table 7). The HD group showed significantly higher Observed_species, Shannon, and Simpson indices compared to the CK and LP groups (*p* < 0.05), while no significant differences were observed between the CK and LP groups. Notably, the CK group exhibited the highest Chao1 and ACE indices, reflecting distinct patterns in species richness estimation. These results suggest that LP and HD feed modulates intestinal microbiota by enhancing microbial diversity (Shannon: 4.12 in CK vs. 4.27 in LP vs. 4.79 in HD) while maintaining comparable species richness to basal feed.

As illustrated in the PCoA plot (Unweighted Unifrac distance), each point represents an individual abalone sample, with the color corresponding to experimental groups (HD, LP, CK). The analysis revealed distinct clustering patterns: samples within the same group exhibited tight aggregation, while intergroup points were spatially separated without overlap. This clear segregation (PERMANOVA, * *p* < 0.001) indicates that dietary interventions significantly restructured the intestinal microbial community composition of abalone.

#### 3.6.2. Effects of Fermented Feed on Gut Microbiota Composition in Abalone

Following species annotation processing, a comparative analysis of microbial communities was conducted based on relative abundance at both the phylum and genus levels. A total of 37 bacterial phyla and 1156 bacterial genera were identified across all intestinal samples. Based on annotation results, the top 10 phyla accounting for over 99% of total annotated species in abalone gut microbiota from different marine areas were selected for analysis.

Figure 4 illustrates the phylum-level effects of fermented feed on abalone gut microbiota. The dominant phyla included Proteobacteria, Tenericutes, Bacteroidetes, Firmicutes, Actinobacteria, Planctomycetes, and Verrucomicrobia. Significant intergroup differences were observed in Acidobacteria, Spirochaetes, and Chloroflexi (*p* < 0.05).

Notably, Proteobacteria showed no significant variation among the experimental groups. The LP group exhibited the significant upregulation of Tenericutes (*p* = 0.017) and downregulation of Bacteroidetes (*p* = 0.023) compared to controls. Although Firmicutes abundance showed no statistical difference from the CK group, it was significantly lower than the HD group (*p* = 0.035).

At the genus level (Figure 4C), the dominant genera comprised *Curvibacter*, *Mycoplasma*, *Asinibacterium*, *Mycoplasmopsis*, *Ochrobactrum*, *Pandoraea*, *Hydrotalea*, and *Pelagibacterium*. The LP group demonstrated 3.08-fold and 14.42-fold increases in *Mycoplasma* and *Ochrobactrum* abundance, respectively, versus CK group, concomitant with 2.35-fold reduction in *Pelagibacterium*. *Curvibacter*, *Asinibacterium*, *Pandoraea*, and *Hydrotalea* showed no significant differences from the CK group, but were notably reduced compared to the HD group.

Linear discriminant analysis (LDA > 4) revealed 31 differentially abundant species across groups (Figure 4(A3)). The CK, HD, and LP groups contained seven, fourteen, and ten discriminant species, respectively, contributing to microbial structure variation. Notably, *Lactobacillus plantarum*-fermented feed enhanced differential microbiota diversity in H. discus hannai compared to the CK group. Although the LP group showed slightly lower diversity than the seaweed-fed HD group, its increased discriminant species count relative to the CK group indicates probiotic-fermented feed’s modulatory effect on intestinal microbial diversity.

### 3.7. Metabolite Identification and Quantification

**Figure 5:**
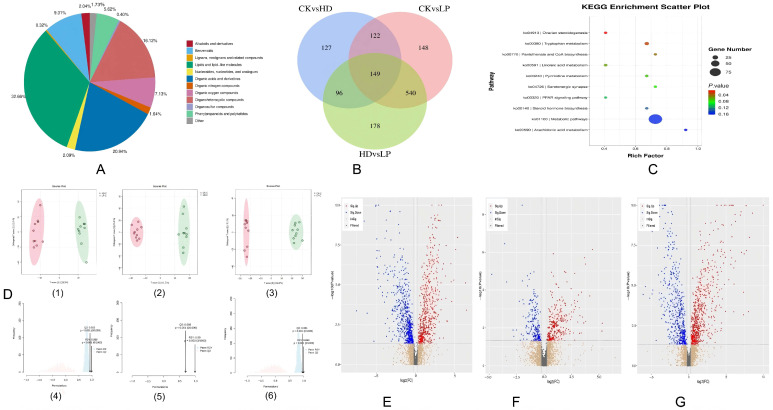
Effects of fermented feed on metabolites composition in abalone gut. (**A**) Donut plot of metabolite classification and proportion. (**B**) Venn diagram of differential metabolites in each comparison group. (**C**) KEGG diagram of differential metabolic pathways in each comparison group. (**D1**–**D6**) OPLS-DA score diagram of different comparison groups. (**E**–**G**) Differential metabolite volcano plot.

A total of 2478 compounds were identified through LC-MS-based metabolomic profiling combined with an in-house database. The annotated metabolites were classified into 14 major categories (Figure 5): lipids and lipid-like molecules (877), organoheterocyclic compounds (391), organic oxides (167), organic acids and derivatives (460), organohalides (3), organosulfur compounds (11), organonitrogen compounds (52), 1,3-dipolar organic compounds (1), homogeneous non-metallic compounds (1), hydrocarbons and derivatives (7), alkaloids and derivatives (55), lignans/neolignans (8), nucleosides/nucleotides/analogs (52), benzenoids (243), phenylpropanoids/polyketides (120), and others (30).

#### 3.7.1. OPLS-DA Modeling

Untargeted LC-MS/MS metabolomics was performed on ten intestinal samples per group. Orthogonal Partial Least Squares Discriminant Analysis (OPLS-DA) with 1000 permutation tests (to mitigate overfitting risks) revealed distinct clustering patterns among the three groups (Figure 4B). The clear spatial separation (R2Y > 0.98 for all pairwise comparisons) and absence of intergroup overlap confirmed significant metabolic profile differences. Model validity was further supported by high predictive capacity: CK vs. HD (R2Y = 0.99, Q2 = 0.693), CK vs. LP (R2Y = 0.993, Q2 = 0.951), and HD vs. LP (R2Y = 0.985, Q2 = 0.922). All Q2 values exceeded the 0.5 threshold, confirming robust model reliability (Figure 4C).

#### 3.7.2. Screening of Differential Metabolites

Differential metabolites were identified using Variable Importance in Projection (VIP) scores from the first principal component of Orthogonal Partial Least Squares-Discriminant Analysis (OPLS-DA) models combined with independent *t*-test *p*-values. Among the 3254 detected metabolites, 959 showed significant intergroup variations (VIP > 1.0, *p* < 0.05), including 664 upregulated and 405 downregulated compounds in LP compared to CK (Table 8). The key metabolic pathways impacted by fermented feed included lipid biosynthesis (23.1%), amino acid metabolism (18.7%), and secondary metabolite production (14.5%).

#### 3.7.3. Classification of Differential Metabolites

In the CK vs. HD comparison, 494 differential metabolites were identified, including 167 lipids and lipid-like molecules, 96 organoheterocyclic compounds, 35 organic oxides, 93 organic acids/derivatives, 1 organohalide, 2 organosulfur compounds, 11 organonitrogen compounds, 2 hydrocarbons, 14 alkaloids/derivatives, 1 lignan/neolignan, 12 nucleosides/nucleotides/analogs, 32 benzenoids, 24 phenylpropanoids/polyketides, and 4 others, with 286 upregulated and 208 downregulated metabolites.

The CK vs. LP comparison revealed 959 differential metabolites: 346 lipids/lipid-like molecules, 185 organoheterocyclic compounds, 68 organic oxides, 153 organic acids/derivatives, 3 organohalides, 4 organosulfur compounds, 14 organonitrogen compounds, 2 hydrocarbons, 18 alkaloids/derivatives, 4 lignans/neolignans, 18 nucleosides/nucleotides/analogs, 89 benzenoids, 46 phenylpropanoids/polyketides, and 9 others, showing 664 upregulated and 405 downregulated species.

For HD vs. LP, 963 differential metabolites were detected: 355 lipids/lipid-like molecules, 173 organoheterocyclic compounds, 65 organic oxides, 155 organic acids/derivatives, 2 organohalides, 5 organosulfur compounds, 13 organonitrogen compounds, 4 hydrocarbons, 15 alkaloids/derivatives, 2 lignans/neolignans, 1 homogeneous non-metallic compound, 17 nucleosides/nucleotides/analogs, 99 benzenoids, 50 phenylpropanoids/polyketides, and 7 others, with 460 upregulated and 503 downregulated metabolites.

Cross-group analysis identified lipids/lipid-like molecules, organoheterocyclic compounds, organic acids/derivatives, and benzenoids as the predominant differential metabolite categories. A core set of 149 shared differential metabolites was observed across all comparisons (Figure 5).

#### 3.7.4. KEGG Pathway Enrichment Analysis

KEGG pathway analysis (FDR-adjusted *p* < 0.05) revealed the significant enrichment of differential metabolites in ovarian steroidogenesis (*p* = 0.018), tryptophan metabolism (*p* = 0.026), and pantothenate/CoA biosynthesis (*p* = 0.042) (Figure 4). These pathways are hypothesized to regulate growth performance and immune responses in H. discus hannai.

#### 3.7.5. Shared Differential Metabolites Across Groups

Fourteen high-confidence differential metabolites (VIP > 1.0, *p* < 0.05) were identified between the CK and experimental groups (HD/LP) after filtering unnamed compounds (Table 9). Three metabolites were downregulated in the LP/HD groups: cis-11,14,17-eicosatrienoic acid, thermozymocidin, and nabilone. Eleven metabolites exhibited upregulated trends, including the key intermediates of lipid remodeling (lysophosphatidylcholine 18:1) and amino acid derivatives (N-acetylglutamine).

### 3.8. Correlation Analysis of Differential Microbial Genera, Metabolites, and Production Performance in H. discus hannai

**Figure 6:**
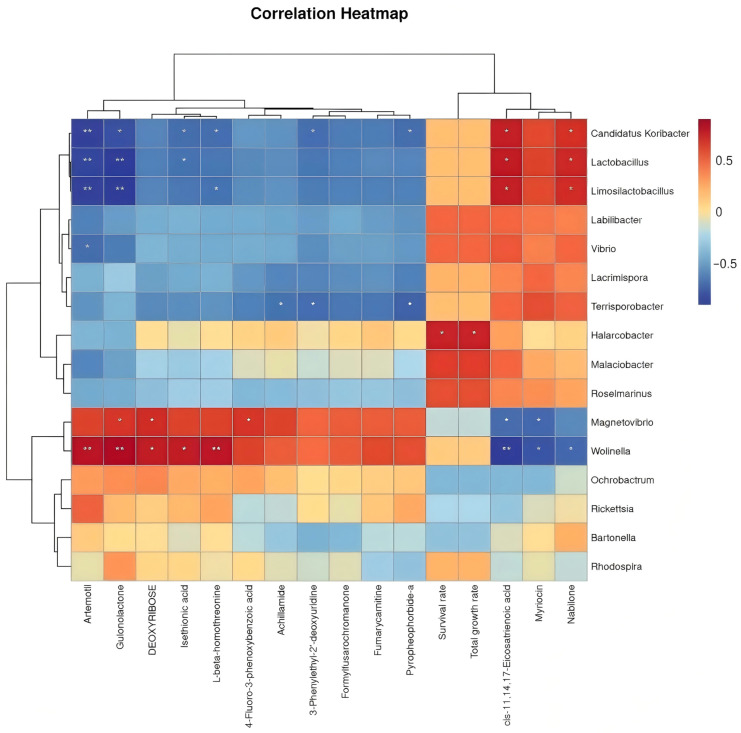
Correlation analysis of differential bacterial genera with other indexes. * *p* < 0.05, ** *p* < 0.01.

To elucidate the potential mechanisms by which LP-fermented feed modulates abalone production performance through intestinal microbiota–metabolite crosstalk, a Spearman correlation analysis was conducted between the differential bacterial genera, metabolites, growth indices (total weight gain rate), and survival rate. The association heatmap (Figure 6) reveals distinct interaction patterns, where the red and blue regions denote positive and negative correlations, respectively, reflecting the potential microbial regulation of metabolic pathways and host physiology.

## 4. Discussion

This study multidimensionally evaluates the effects of soybean meal fermented with Lactobacillus plantarum (LP) on feed quality, growth performance, intestinal health, and metabolic regulation in abalone (H. discus hannai). The results demonstrate that LP fermentation significantly enhanced key fatty acids in soybean meal, including a two-fold increase in cis-9-palmitoleic acid and a 14% elevation in α-linolenic acid (ALA), alongside notable improvements in amino acids such as L-histidine and L-asparagine.

Critically, these nutritional enhancements translated to significant biological outcomes. Feeding with LP-fermented feed markedly improved abalone growth rate (263% vs. 201% in the control group, CK) and survival rate (61.66% vs. 45.33% in CK). Beyond growth metrics, mechanistic investigations revealed profound effects on intestinal physiology: LP fermentation enhanced intestinal morphology (elongated villi and structural integrity), upregulated tight junction proteins (Claudin and ZO-1), suppressed inflammatory cytokines (TNF-α, IL-16), and activated antioxidant pathways (Keap1 downregulation), collectively optimizing intestinal health.

Further elucidating the drivers of improved health and growth, LP-fed abalone exhibited increased gut microbiota diversity, elevated abundances of Mycoplasma and Ochrobactrum, and differential metabolites enriched in tryptophan metabolism and the pantothenate/CoA biosynthesis pathways, revealing a potential microbiota–metabolism axis driving growth promotion.

Positioning these findings within existing knowledge, this study aligns with prior research on probiotic-fermented feeds in aquaculture, but also unveils novel insights. Regarding nutritional enhancement, earlier studies consistently reported that microbial fermentation degrades antinutritional factors in soybean meal and liberates free amino acids. For instance, N. Muhamad Nor [21] observed significant increases in lysine and methionine content after lactic acid bacteria fermentation, consistent with the marked elevation of L-histidine and L-asparagine here. However, our work uniquely highlights the paradoxical enrichment of ω-3 fatty acids (ALA) alongside DHA reduction—a phenomenon seldom reported. This discrepancy may stem from LP’s metabolic specificity: earlier studies suggested that lactic acid bacteria preferentially degrade long-chain fatty acids via β-oxidation [22], potentially accelerating DHA catabolism, while ALA accumulation could arise from bacterial biosynthesis or selective substrate utilization [23]. These nuanced findings underscore the need for multi-omics approaches (e.g., lipidomics and transcriptomics) to dissect fatty acid metabolic networks.

Transitioning to growth performance, while LP-fed abalone exhibited higher weight gain than CK, it underperformed compared to the seaweed-based HD group—a result partially congruent with [24], who demonstrated that fermented soybean meal could replace 30% of fishmeal without compromising fish growth. The likely explanation for HD’s superiority resides in its polysaccharide-rich matrix and trace element content, unmatched by fermented feeds. Notably, a key advantage of the LP diet emerged in efficiency: the significantly lower feed conversion ratio (FCR) implies enhanced nutrient utilization, a mechanism plausibly linked to the observed intestinal remodeling and microbiota modulation. For example, villus elongation directly expands nutrient absorption surfaces [25], while upregulated tight junction proteins fortify barrier integrity, mitigating leaky gut risks [26]. These observations not only corroborate the “gut health–nutrient utilization” axis proposed in probiotic studies, but extend it by integrating structural, immunological, and microbial dimensions—a holistic perspective that was previously absent.

The gut microbiota findings further reveal both consonance and divergence from the literature. The marked enrichment of Tenericutes and Mycoplasma in LP-fed abalone contrasts with Ifra Ghori et al. (2022) [27], who reported the probiotic-driven suppression of conditional pathogens in fish. Significantly, the 14-fold surge in Ochrobactrum—a genus renowned for xenobiotic degradation [28]—suggests its role in detoxifying feed-derived contaminants, indirectly bolstering host health. Nevertheless, comparative analysis highlights a challenge: the higher OTU diversity in the HD group underscores the ecological superiority of natural diets in maintaining microbial stability, urging the future exploration of hybrid feeding strategies.

Metabolomic insights add another layer of understanding, delineating the multidimensional regulatory effects of LP fermentation. Differential metabolites enriched in the tryptophan metabolism (kynurenine pathway) align with suppressed TLR4/NF-κB signaling, implying that tryptophan derivatives mitigate intestinal inflammation—a mechanism known in mammals [29], now extended to mollusks. Crucially, the upregulation of artemotil and isethionic acid, correlated with Wolinella abundance and growth metrics, provides direct evidence for a “microbiota–metabolite–host” axis. This multi-omics integration transcends conventional single-parameter analyses, offering unprecedented resolution into probiotic mechanisms—an advancement addressing critical gaps in earlier aquaculture research.

Finally, this study identifies some key limitations that point to future directions: the metabolic mechanism driving DHA reduction remains unclear, potentially addressable by stable isotope tracing; the absence of long-term assessments on reproductive performance necessitates extended trials; and the lack of functional gut microbiota profiling (metagenomics) limits causal insights into microbiota–metabolite interactions, highlighting the need to integrate metagenomics with metabolic flux studies. And the potential for enhanced resistance against specific pathogens like Vibrio harveyi requires validation through dedicated challenge trials. Additionally, exploring yeast co-fermentation strategies may further optimize fatty acid composition and feed’s nutritional value.

## 5. Conclusions

This study multidimensionally deciphers how LP-fermented soybean meal promotes abalone growth through three synergistic mechanisms: nutrient fortification, intestinal health optimization, and microbiota–metabolome network remodeling. These findings advance the theoretical framework for probiotic applications in aquaculture by establishing quantitative correlations between fermentation-induced nutritional shifts (ALA accumulation) and host physiological responses. Specifically, this study provides actionable insights for sustainable feed development, including optimizing fermentation parameters to enhance GABA production for improved stress resistance and modulating microbiota-metabolite crosstalk to promote growth efficiency.

## Figures and Tables

**Table 1:** Effects of LP-fermented soybean meal on sensory parameters.

Group	Color	Odor	Texture	Agglomeration	pH
CK	Light yellow	Bean cake aroma	Firm	Loose granules	6.5
LP	Brownish-yellow	Sour alcoholic aroma	Soft	Agglomerated	6.7

CK: control group; LP: LP-fermented group.

**Table 2:** Composition of unsaturated fatty acids in soybean meal (mg/kg).

Free Fatty Acid	CK	LP	*p*-Value
Trans-9-myristoleic acid	0.2 ± 0.04	0.1 ± 0.02	0.063
Trans-9-palmitoleic acid	1.1 ± 0.18	2.38 ± 0.33	0.082
Cis-9-palmitoleic acid	48.32 ± 9.18	106.85 ± 21.37	0.023
Trans-9-octadecenoic acid	3.08 ± 0.46	3.7 ± 0.7	0.079
Cis-11-octadecenoic acid	122.78 ± 18.42	124.87 ± 24.97	0.057
Cis-13-erucic acid	5.35 ± 0.75	5.42 ± 0.87	0.065
Trans-13-docosenoic acid	3.49 ± 0.45	3.62 ± 0.36	0.073
Cis-15-nervonic acid	3.11 ± 0.4	3.42 ± 0.55	0.056
A-linolenic acid (ala)	256.6 ± 33.36	292.6 ± 55.59	0.017
Cis-11,14,17-eicosatrienoic acid	1.97 ± 0.28	2.47 ± 0.3	0.045
Cis-4,7,10,13,16,19-docosahexaenoic acid (dha)	75.33 ± 10.55	66.47 ± 13.29	0.014
Cis-9,12-linoleic acid (la)	23.58 ± 4.24	23.27 ± 3.72	0.061
Γ-linolenic acid (gla)	2.57 ± 0.28	2.33 ± 0.33	0.078
Cis-8,11,14-dihomo-γ-linolenic acid (dgla)	2.44 ± 0.49	2.47 ± 0.47	0.089
Arachidonic acid (aa)	23.56 ± 3.53	21.48 ± 2.79	0.051
Cis-7,10,13,16-docosatetraenoic acid	4.53 ± 0.91	4.72 ± 0.52	0.091

Data presented as mean ± standard deviation. CK: control group; LP: LP-fermented group.

**Table 3:** Composition of saturated fatty acids in soybean meal (mg/kg).

Free Fatty Acid	CK	LP	*p*-Value
Caprylic acid (C8:0)	3.41 ± 0.55	2.71 ± 0.33	0.013
Lauric acid (C12:0)	0.21 ± 0.03	0.96 ± 0.11	0.011
Tridecanoic acid (C13:0)	0.11 ± 0.01	0.29 ± 0.04	0.032
Myristic acid (C14:0)	30.34 ± 4.25	45.94 ± 9.19	0.017
Pentadecanoic acid (C15:0)	9.31 ± 1.58	13.28 ± 2.66	0.025
Palmitic acid (C16:0)	1520.93 ± 273.77	1685.45 ± 219.11	0.043
Heptadecanoic acid (C17:0)	18.18 ± 3.45	21.92 ± 3.29	0.041
Stearic acid (C18:0)	345.27 ± 65.6	350.38 ± 63.07	0.069

Data presented as mean ± standard deviation. ND: not detected; CK: control group; LP: LP-fermented group.

**Table 4:** Effects of probiotic additives on amino acid content in feed before and after fermentation (g/kg).

Amino Acid	CK	LP	*p*-Value
β-Alanine	0.26 ± 0.01	0.32 ± 0.01	0.045
γ-Aminobutyric acid (GABA)	14.72 ± 0.48	19.56 ± 0.41	0.021
L-Proline	8.68 ± 0.08	9.35 ± 0.13	0.056
L-Asparagine	73.43 ± 2.57	99.94 ± 6.77	0.023
L-Glutamine	10.09 ± 0.35	12.79 ± 0.78	0.062
L-Methionine	46.28 ± 0.86	51.73 ± 0.39	0.051
L-Histidine	9.77 ± 0.09	13.55 ± 0.4	0.008
3-Methyl-L-histidine	0.41 ± 0.01	0.49 ± 0.01	0.078
1-Methyl-L-histidine	0.20 ± 0.01	0.23 ± 0.01	0.098
L-Arginine	23.21 ± 0.13	30.28 ± 0.58	0.015
L-Tryptophan	7.54 ± 0.24	7.81 ± 0.16	0.049

Data presented as mean ± standard deviation. CK: control group; LP: LP-fermented group.

**Table 5:** Growth performance indexes of *H. discus hannai*.

Group	HD	CK	LP	*p*-Value
Total weight gain rate (%)	421%	201%	263%	0.017
Survival rate (%)	69%	45.33%	61.66%	0.031
Daily weight gain rate (%)	19%	9.5%	12.44%	0.018
Feed conversion ratio (FCR)	2633.12	1586.37	1205.36	0.014

Data presented as mean ± standard deviation. HD: natural feed group; CK: control group; LP: LP-fermented group.

**Table 6:** Effects of different diets on amino acid content of abalone muscle quality.

Amino Acid g/KG	HD	LP	CK	*p*-Value
Aspartic acid	1.21 ± 0.12	1.26 ± 0.16	1.19 ± 0.12	0.058
Threonine	0.53 ± 0.08	0.57 ± 0.14	0.56 ± 0.16	0.121
Serine	0.62 ± 0.12	0.62 ± 0.15	0.54 ± 0.07	0.087
Glutamic acid	2.04 ± 0.55	2.1 ± 0.55	2.03 ± 0.26	0.042
Glycine	1.15 ± 0.12	1.02 ± 0.29	0.97 ± 0.16	0.036
Alanine	0.77 ± 0.2	0.76 ± 0.15	0.72 ± 0.2	0.231
Cystine	0.01 ± 0	0.11 ± 0.02	0.09 ± 0.01	0.011
Lavandulin	0.51 ± 0.14	0.55 ± 0.1	0.46 ± 0.12	0.063
Methionine	0.29 ± 0.07	0.3 ± 0.03	0.25 ± 0.04	0.079
Isoleucine	0.47 ± 0.08	0.51 ± 0.13	0.47 ± 0.12	0.092
Leucine	0.82 ± 0.19	0.89 ± 0.24	0.88 ± 0.17	0.152
Tyrosine	0.36 ± 0.1	0.4 ± 0.09	0.34 ± 0.07	0.035
Phenylalanine	0.37 ± 0.1	0.41 ± 0.09	0.32 ± 0.04	0.012
Lysine	0.72 ± 0.07	0.82 ± 0.22	0.76 ± 0.21	0.055
Histidine	0.18 ± 0.03	0.21 ± 0.06	0.11 ± 0.02	0.034
Arginine	1.31 ± 0.3	1.29 ± 0.35	1.21 ± 0.3	0.048
Proline	0.55 ± 0.09	0.5 ± 0.07	0.44 ± 0.04	0.053

Data presented as mean ± standard deviation. HD: natural feed group; CK: control group; LP: LP-fermented group.

**Table 7:** Alpha diversity indices of intestinal microbiota in H. discus hannai.

Group	Observed Species	Chao1	ACE	Shannon	Simpson
CK	2413.40	4580.73	5097.43	4.12	0.89
LP	2358.60	2886.44	3168.66	4.27	0.89
HD	2916.10	3905.55	4343.13	4.79	0.92
*p*-value	0.047	0.015	0.027	0.032	0.159

HD: natural feed group; CK: control group; LP: LP-fermented group.

**Table 8:** Comparative analysis of differential metabolites across experimental groups.

Comparison Group	Total Differential Metabolites (Diff_All)	Upregulated Metabolites (Diff_Up)	Downregulated Metabolites (Diff_Down)
CK vs. HD	494	286	208
CK vs. LP	959	664	405
HD vs. LP	963	460	503

HD: natural feed group; CK: control group; LP: LP-fermented group.

**Table 9:** High-confidence differential metabolites.

Metabolite Name	log2 FC		*p*-Value		VIP Value		Trend
	HD vs. CK	LP vs. CK	HD vs. CK	LP vs. CK	HD vs. CK	LP vs. CK	
Artemether	0.98	2.98	<0.05	<0.01	1.29	1.90	↑
Pyropheophorbide A	2.00	6.93	<0.01	<0.01	1.97	1.90	↑
cis-11,14,17-Eicosatrienoic acid	−1.12	−2.59	<0.05	<0.01	1.22	1.66	↓
Fumaroylcarnitine	0.81	2.81	<0.01	<0.01	1.69	1.86	↑
Formylfuranobenzopyrone	1.31	6.28	<0.03	<0.01	1.43	1.90	↑
Thermocidin	−0.56	−3.26	<0.01	<0.01	2.09	1.93	↓
L-β-Homoserine	0.59	1.57	<0.01	<0.01	1.96	1.84	↑
Azithromide	0.91	4.37	<0.01	<0.01	1.98	1.81	↑
Hydroxyethylsulfonic acid	0.87	5.97	<0.01	<0.01	1.86	1.96	↑
D-Gulonic acid gamma-lactone	1.03	4.77	<0.01	<0.01	2.06	1.96	↑
2-Deoxy-beta-L-erythro-pentafuranose	1.15	4.35	<0.01	<0.01	2.32	1.95	↑
Nabilone	−1.52	−3.90	<0.01	<0.01	2.36	1.88	↓
3-Phenethyl-2′-deoxyuridine	2.13	10.88	<0.01	<0.01	1.77	1.95	↑
4-Fluoro-3-phenoxybenzoic acid	3.70	5.81	<0.01	<0.01	2.53	1.91	↑

↑ indicates upregulation, ↓ indicates downregulation HD vs. CK: High Dose vs. Control. LP vs. CK: Low Dose vs. Control; FC: Fold Change; VIP: Variable Importance in Projection. Note: Some metabolite names have been translated using standard IUPAC nomenclature and common biochemical naming conventions.

## Data Availability

The datasets presented in this study can be found in online repositories.
